# In Situ Analysis of Electrodermal Activity from Students Learning from Large Language Models Versus Curated Texts

**DOI:** 10.3390/brainsci16020153

**Published:** 2026-01-29

**Authors:** Kenneth Y. T. Lim, Yue Heng Wong, Duc Nam Tran, Edrik K. X. Lee, Thien Minh Tuan Nguyen, Duc Minh Anh Nguyen, Alan J. H. Tan

**Affiliations:** 1 National Institute of Education, 1 Nanyang Walk, Singapore 637616, Singapore; 2 Independent Scholar, Singapore 637616, Singaporenguyenthienminhtuan526705@gmail.com (T.M.T.N.); anhnguyenducminh@gmail.com (D.M.A.N.);

**Keywords:** science of learning, neuroergonomics, electrodermal activity, wearables, Large Language Models, Generative Artificial Intelligence

## Abstract

**Highlights:**

**What are the main findings?**

- Generative AI and the use of Large Language Models have varying degrees of receptivity and impact in teaching and learning contexts.
- In this nascent field, studies have so far focused on medium-term usage patterns and their possible consequences.

**What are the implications of the main findings?**

- This paper investigates the affective states of learners in situ as they are interacting with Large Language Models.
- The granularity of the in situ data differs from those obtained through *post hoc* means from prior work and offers insight into the efficacy (or lack thereof) of the use of Large Language Models in the arousal levels of learners.
- Implications for practice and/or policy: The use of low-cost, non-invasive wearables (which have been validated against industry-grade equipment) in the study suggests possibilities for future work to scale the investigation to other socio-cultural contexts of learning.

**Abstract:**

Background: this paper reports an investigation into the cognitive and emotional states of adolescents while learning from an LLM. It seeks to address a relative dearth in empirical evidence which might otherwise facilitate informed decisions being made by curriculum designers, school leaders and policy makers regarding the use of Generative AI, amidst the wider discourse about the effectiveness of AI in teaching and learning. Methods: in this paper, we analyze electrodermal activity (EDA) in the context of students’ scholastic engagement using LLMs in comparison to curated texts. In our 27-min-long experiment, we recorded the EDA of participants learning from both learning methods, for 8 min each. A quiz was also conducted to assess the effectiveness of the learning method. We collected 23 samples of EDA from the experiment, and 42 samples of quiz results. Results: we have found that learning with an LLM results in greater Skin Conductance Response (*p* = 0.09404), which is linked to more positive emotional valence, and lower Skin Conductance Level (*p* = 0.09473), which is linked to lower cognitive load, compared to curated texts. We also discovered that learning with an LLM correlates to a higher quiz result (*p* = 0.02053). While this suggests that learning and absorbing information with an LLM could be more effective than curated texts, results from self-reported data indicate that there are few perceived differences between the effectiveness of LLM and curated texts. Conclusions: this exploratory and preliminary study revealed empirical insights between LLM usage and learning effectiveness in situ via physiological indicators, in contrast to prior work that has adopted *post hoc* frames over the medium- to long-term.

## 1. Introduction

Understanding how different types of instructional content impact student learning and arousal is critical in this era of advanced artificial intelligence (AI). In recent years, Large Language Models (LLMs) such as ChatGPT have prompted critical debate on the way educational content is generated and consumed [1]. These models can produce extensive, coherent, and contextually relevant text, raising questions about their effectiveness compared to traditional, human-written educational materials.

Prior studies have highlighted the potential of LLMs to provide tailored learning experiences for individual students, allowing students to participate in a curriculum adaptive to their respective paces of learning [2]. Other studies highlighted the potential of LLMs in aiding problem solving, critical thinking and motivation of students, as demonstrated by Vazquez-Cano et al. (2021) and Kim and Lee (2023) [3,4]. However, the discourse on the effectiveness of Generative AI also brought opposing notions. According to a study in 2025 by Gerlich, it was proposed that as people rely more on AI technology and for an extended time period, they are less likely to engage in critical thinking as they could resort to AI for cognitively straining tasks [5]. This behavior—referred to as cognitive offloading—is detrimental and ineffective in boosting learners’ efficacy. Another study by Abbas et al. (2024) [6] also corroborated this effect of Generative AI. It stated that students who face higher pressure (time, academic workload) were more likely to rely on AI [6]. Subsequently, prolonged and repeated usage and reliance on AI led to memory loss and delay of work. This two-sided argument is aptly summarized in a meta-analysis by Huang et al., which stated that the effects of LLMs on students can be positive, although uneven and applied on a small scale. Furthermore, the study also highlights that the nature of these effects depends heavily on how student agency and participation are scaffolded [7]. The ongoing discourse and vast parameters further highlight the need for empirical evidence on the effectiveness of AI and LLMs.

Most studies on this topic study the effect of LLMs on learning over long periods of time (e.g., over months at a time) through *post hoc* means. As seen in previous works, longitudinal studies are primarily focused on presenting cumulative effects of studying using AI, for example, cognitive offloading. In contrast, there exists a relative dearth of research on in situ measures of the effect of LLMs on learning performance. Studying the effect of LLMs in the short term opens new avenues for research and explanation, as more data can be collected from participants. Most notably, short-term studies can allow for research on more granular interactions between education and AI with suitable control of external factors. This, in turn, can complement what is suggested by existing studies on the long-term usage of LLMs in learning new knowledge. To summarize, within this context, while longitudinal studies confirm broader trends and the effects of reliance on AI, short-term studies provide granular, context-specific insights through case studies that maintain verisimilitude with real-life scenarios, which longitudinal studies fail to capture (e.g., short-term/one-time querying about a topic or new information). Within these parameters, this study also adopts a distinct approach by incorporating biometric data.

Another aspect of interest is the link between emotions and learning. It is widely known that affective states have a significant influence on efficiency, productivity and behaviors. Learning is no different, as emotions are intertwined with every aspect of the learning process [8]. To elaborate on this, positive emotions, such as joy, encourage the learning process, while negative emotions, such as fear, inhibit the learning process [9]. As such, an investigation into the emotions that occur during learning from LLMs could allow us to have a deeper understanding of the learning process when using LLMs. With regards to this, Russel’s model can give a general estimate of affective state using a two-dimensional scale of emotional arousal and valence [10]. In our experiment, we assume affective state to be the marker of the effectiveness of obtaining knowledge via LLM and curated text.

In the study, we investigate the near-term effects of LLMs on learning performance via the analysis of Electrodermal Activity (EDA). EDA, which measures the electrical conductance of the skin in response to sweat secretion, is intricately linked to the sympathetic nervous system and offers valuable data on mental and physiological states [11]. Hence, EDA is an important tool for assessing physiological responses due to its ability to provide non-invasive insights into human stress and emotional arousal. This provides almost real-time insights into the cognitive and emotional stresses of learning during the short term. Compounded with the fact that students are more likely to react strongly to new information for the first time, more rapid fluctuations in EDA peaks and troughs are likely to be captured. By capitalizing on this, short-term studies can provide more meaningful empirical evidence, which longitudinal studies fail to capture as students gradually become more acclimatized to LLM and the subjects at hand.

In summary, the rapid adoption of AI-generated content in education necessitates a deeper understanding of its impact on students’ cognitive and emotional states. By using EDA as a non-invasive method to monitor students’ stress and arousal during learning activities, this research aims to provide empirical data on how AI-generated texts compare to human-written texts.

This research involves the use of low-cost, wearable devices, such as a biometric wristband, to measure EDA alongside other physiological indicators in students. These devices have been validated against research-grade equipment to ensure accuracy and reliability [12]. The research involves a controlled experiment where students engage with educational content produced both by LLM and human authors, allowing for a direct comparison between learning from AI-generated texts and human-written texts. This approach allows for a nuanced analysis of the interplay between educational content and student arousal.

Our research seeks to demonstrate the potential of EDA data in enhancing our understanding of educational practices. By comparing the physiological impacts of AI-generated and human-written texts, we aim to contribute valuable insights that can inform the design of future educational tools and strategies. The findings could have significant implications for the integration of AI in educational settings, ultimately leading to improved learning experiences for students.

## 2. Background

Critchley and Nagai (2012) [13] define electrodermal activity (EDA) as changes in skin resistance or electrical potential due to neural effects on sweat glands. Comprising tonic (skin conductance level, SCL) and phasic (skin conductance response, SCR) elements, EDA is closely linked to human stress and emotions, and can be measured non-intrusively [11]. This makes it a practical tool for assessing stress and emotions outside of laboratory settings.

On the other hand, according to a study by Leblanc and Posner in 2022, learning consists of both cognitive factors and emotional ones [14]. Important factors in learning, such as attention [15] and memory [16], have shown ties to emotional variations. Given that both EDA and learning have deep correlations to the same physiological factors, it is likely that a relationship exists between the two.

Analysis of an EDA signal requires the calculation of certain key features. In this study, we use a wide range of features in order to study potential differences in EDA across various learning methods.

This study used the number of non-specific skin conductance responses (NSSCR) per minute, as previous research links NSSCR increases to emotional arousal [17] and, when combined with negative valence, to emotional stress [18].

The study also included Mean Skin Conductance Level (SCL) and Skin Conductance Response (SCR), which reflect tonic and phasic changes in skin conductance, respectively. Research has found that SCL is an indicator of cognitive load [19], while the amplitude of SCR tends to rise with increased emotional valence when individuals view visual stimuli [20].

TVSymp is a sensitive metric of sympathetic activity derived from time-frequency spectral analysis of EDA data [21], and has demonstrated high sensitivity to orthostatic, cognitive, and physical stress [22]. EDASymp, based on power spectral analysis [23], also effectively detects stress, which is comparable to traditional time-domain measures like SCL and NSSCR.

Based on the preceding arguments, we hypothesize that LLM participants will show higher values for each of the five features (NSSCR, Mean SCL, Mean SCR, TVSymp, EDASymp).

## 3. Methodology

### 3.1. Materials Used

A total of 44 participants were recruited through convenience sampling from the peer network of the authors. The participants were aged 13 to 16 with an equal mix of genders.

Sustainability education was chosen as the topic to be learned via the two learning methods, as it was a topic most participants were not familiar with. In this way, it was less likely that bias could occur if participants had prior knowledge about the topic.

A passage of prose about the topic was prepared without the assistance of Generative AI prior to the experiment. It was prepared in the tone of an academic article. The handout is included as Appendix A.

For AI-generated text, we used ChatGPT 4o API as the LLM with our own custom Web App for easier experimentation. The system prompt given to ChatGPT is included as Appendix B. We used the CO-STAR framework when designing the prompt because the response given by the LLM would be helpful and relevant to the topic [24]. The human-written text was included in the prompt as a guide.

### 3.2. Data Collection

EDA data were collected from participants using DIY sensors designed and assembled based on [25]. The EDA circuit follows DC-EXM (exosomatic methodology), where an external current of 3.3 V is applied for measurement of skin resistance, with a sampling rate of 51.2 Hz. Further information and validation of the sensors against research-grade equipment can be found in [12]. Our design of this DIY EDA device follows the design principle of wearables from the company Shimmer, which has been used in numerous experiments that yielded positive results.

**Figure 1:**
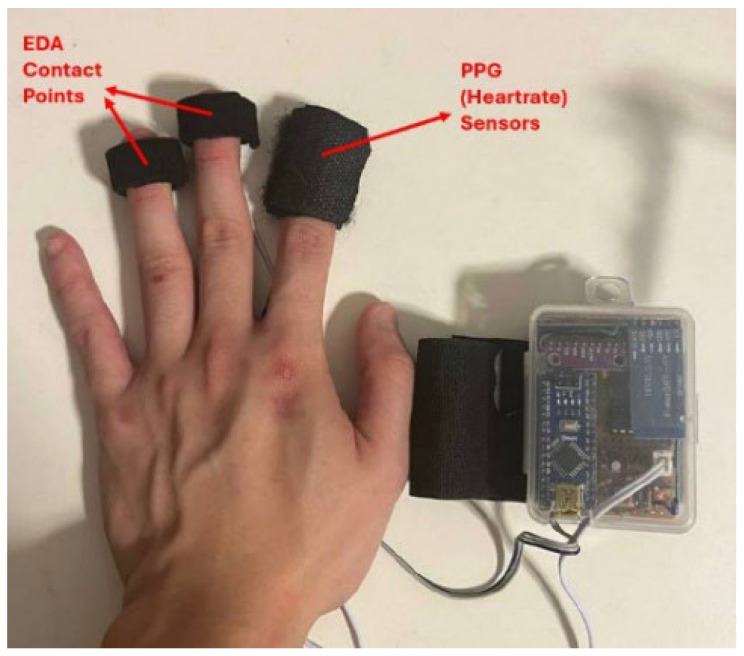
Sensor Set-up.

The sensor was attached to participants’ non-dominant hand, as shown in Figure 1, to allow unrestricted and comfortable movement of the dominant hand during the activities described above. Participants were additionally instructed to minimize movement of the non-dominant hand in order to reduce motion-related data artifacts. This placement was chosen primarily for convenience, as electrical skin conductance is theoretically comparable between both hands. Dry Ag/AgCl electrodes of diameter 0.80 cm were used for the electrodes for measurement of EDA. For the PPG sensor, a pre-assembled model was used (XD-58C) with a sampling rate of 50 Hz.

Data collected during the experiment was split based on the timings outlined in the procedure and analyzed following the processes outlined in the Data Analysis portion below. All data was anonymized, and no personally identifiable information was collected.

In addition to EDA data, we also wished to investigate the efficacy of each learning method, as well as the mental states of the participants after learning from each learning method. A quiz was used to achieve the former, while Self-Assessment Manikin (SAM) evaluations were used to achieve the latter. SAM is a “non-verbal pictorial assessment technique that directly measures the pleasure, arousal, and dominance associated with a person’s affective reaction to a wide variety of stimuli” [26]. SAM was chosen for its effectiveness in directly assessing the pleasure, arousal, and dominance associated with response to the event, which reduces the resources needed to measure other types of variables for higher resolution, according to Bynion and Feldner [27]. Furthermore, SAM can be used universally as language barriers can be overcome when emotions are represented by pictures [28].

To assess participants’ understanding of the topic after learning, a quiz was prepared prior to the experiment. The quiz consisted of six MCQs (Multiple Choice Questions) and a SAM evaluation. The quiz was administered to participants via a physical hardcopy. To guide participants towards getting the correct answers for the MCQs, the scope of the questions was included in both the human-written text handout (Appendix A) and the LLM prompt (Appendix B).

A separate hardcopy was also prepared (SAM evaluation), which only includes the SAM evaluation.

### 3.3. Procedure

**Figure 2:**
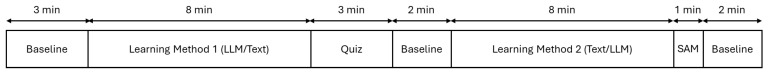
Experiment Timeline.

The flow of the experiment follows the schedule in Figure 2 below.

Sensors were attached to the fingers of the participant’s non-dominant hand before the experiment and removed only after it ended.

The experiment measured baseline EDA at the start, after the quiz, and at the end, alongside EDA during learning. This enabled normalization between participants. Baseline periods ranged from 120 to 180 s (2–3 min).

The experiment includes two 8-min learning windows for participants to learn about the topic via one of the two learning methods (AI-generated text/human-written text). For human-written text, the hardcopy handout (Appendix A) was given out. No annotation was allowed. Participants each used a laptop to interact with the LLM (see Appendix B), asking additional questions as needed. Their EDA was monitored throughout for analysis of changes during learning. Any shift in sentiments between learning methods would then be captured through EDA data and self-reported data through the questionnaires.

All participants had to learn from both methods. The order in which the participants learned from the different learning methods was randomized among participants. While the asymmetry between the LLM-based learning condition and the curated-text condition is acknowledged, this design choice was intentional and aligned with the primary objective of the study, which is to compare traditional self-directed text-based learning with learning mediated by LLMs. Although classroom learning typically allows opportunities for clarification through teacher interaction, replicating such facilitation would introduce additional uncontrolled variables, including variability in question type, timing, relevance across participants, and potential disruptions to reading flow and experimental duration. In the LLM condition, participants were not required to engage in deep or exploration questioning; instead, they retained the option to request information verbatim, closely approximating textbook reading. Any further engagement with the LLM was therefore contingent on individual participant attitudes, behaviors, and emotional states at the time of learning. This design also reflects a realistic self-learning scenario in which students study independently without direct instructional support. Within this context, the study seeks to examine whether access to an LLM confers learning or affective advantages over static text, and to what extent such differences can be quantified and explained using features derived from electrodermal activity (EDA) data.

After the first learning window, the quiz was administered to participants, regardless of the learning method they used. The resultant score of the quiz serves as a measure of participants’ understanding of the topic. This was analyzed to quantify the efficacy of the learning method. The SAM evaluation in the quiz also allowed us to record the participants’ mental and emotional status.

After the second learning window, a second SAM evaluation was performed. This allowed for the analysis of mental and emotional status after each of the learning methods.

### 3.4. Data Analysis

The EDA data was processed in Python 3.12, resampled to 25 Hz, and outliers (absolute z-score > 3) were removed. Data normalization and a low-pass Butterworth filter (1.5 Hz, 8th order) reduced artifacts, or unwanted electronic noise [29]. Further artifact detection and removal followed the LSTM-CNN approach by [30].

The data was segmented into 2–3 min windows matching the baseline length to confirm that EDA feature changes persisted during the learning phase.

Five EDA features were extracted per window: NSSCR/min, SCR, SCL, TVSymp, and EDASympn. NSSCR/min was calculated using a peak detection algorithm from [31]. SCR and SCL were derived via cvxEDA by Greco et al. (2016), separating tonic and phasic components [32]. TVSymp was computed as the mean spectral amplitude in the 0.08–0.24 Hz band using methods from [21]. EDASympn resulted from downsampling to 2 Hz and applying Posada-Quintero et al. (2016b)’s normalized power spectral density approach [23].

Data from the LLM and Text learning phases were normalized by subtracting baseline values. Each feature was tested with a clustered Wilcoxon signed-rank test using the Rosner–Glynn–Lee (RGL) method via the ‘clusrank’ package in R [33], accounting for clustering caused by segmentation.

Data from SAM is compared row-by-row, while data from the quiz is compared directly between the groups. A Wilcoxon signed-rank test was used for both to account for the non-normality of the data. The clustered Wilcoxon test was implemented in R using the clusrank package, specifically employing the Rosner–Glynn–Lee (RGL) method for clustered paired data. Participants were treated as independent clusters, with multiple observations nested within each participant. The RGL method adjusts the variance of the Wilcoxon test statistics to account for within-cluster dependence, thereby providing valid inference under correlated observations. For comparison, standard simple paired Wilcoxon signed-rank tests were additionally conducted as a robustness check. The results of the standard paired Wilcoxon signed-rank tests can be found in Appendix C.

## 4. Results

**Figure 3:**
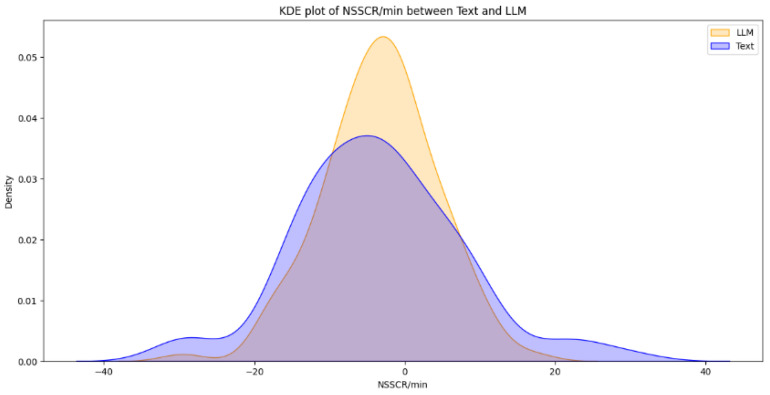
The Kernel Density Estimation plot of NSSCR/minute between Text and LLM.

**Figure 4:**
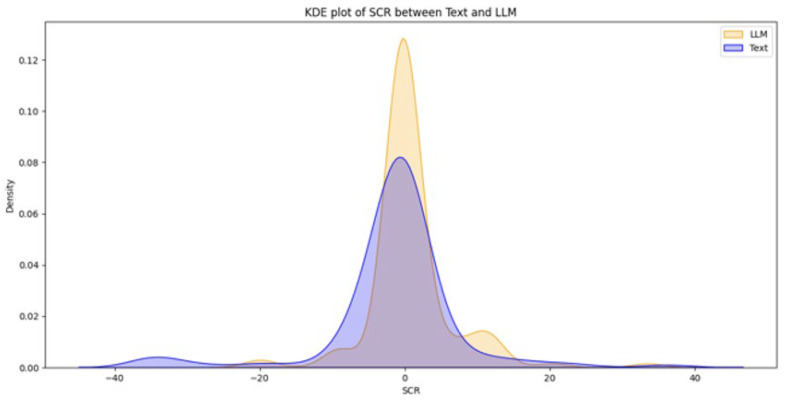
The Kernel Density Estimation plot of SCR between Text and LLM.

**Figure 5:**
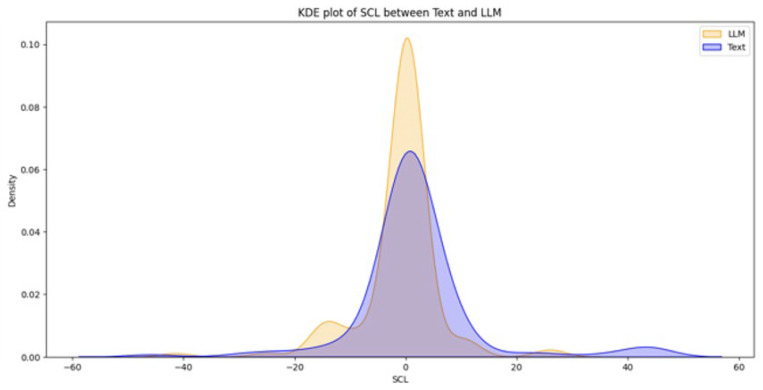
The Kernel Density Estimation plot of SCL between Text and LLM.

**Figure 6:**
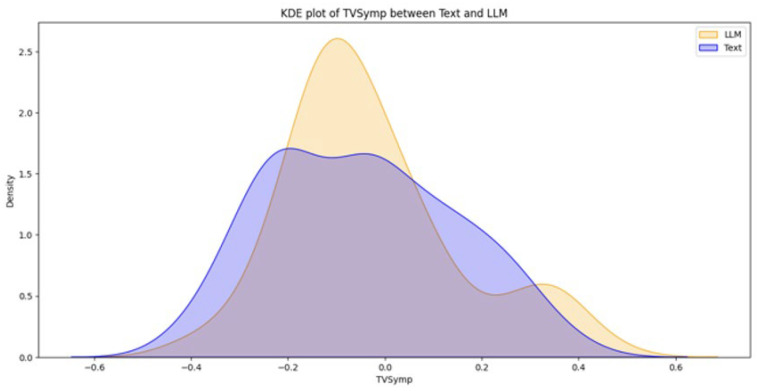
The Kernel Density Estimation plot of TVSymp between Text and LLM.

**Figure 7:**
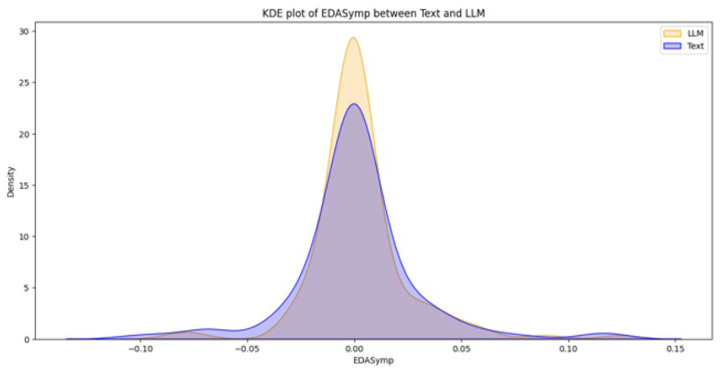
The Kernel Density Estimation plot of EDASymp between Text and LLM.

From a total of 44 participants, the final dataset comprised 23 samples of EDA data (with 21 samples discarded due to hardware faults), 42 samples of quiz results, and 39 samples of SAM evaluations (two samples and five samples discarded, respectively, due to participant incompletion of tasks). Data loss resulted from logistical constraints rather than participant-related factors. Consequently, the missing data are assumed to be missing at random (MAR). Under this assumption, additional sensitivity analyses were not conducted. Figure 3, Figure 4, Figure 5, Figure 6 and Figure 7 below present the respective Kernel Density Estimation plots for each of the five features under investigation, depicting learning from curated text versus learning from Large Language Models.

Since every feature extracted from EDA data collected when participants were learning using an LLM is more right-skewed for everything but SCL, we tested for the alternative hypothesis that the normalized EDA data for LLM is of a greater magnitude than that of Text.

Table 1 depicts how a clustered Wilcoxon signed-rank test showed that individuals using LLM did elicit a statistically significant (*p* < 0.1) increase in normalized SCR in comparison to individuals using Text (Z = 1.3163, *p*-value = 0.09404). Conducting the same test on the rest of the features (NSSCR, TVSymp and EDASymp), the results showed no statistical difference between LLM and curated text, based on the level of significance of *p* < 0.1.

For SCL, we tested for the alternative hypothesis that normalized EDA data for LLM is of a lesser magnitude than that of Text.

A clustered Wilcoxon signed-rank test showed that individuals using LLM did elicit a statistically significant decrease in normalized SCL in comparison to individuals using Text (Z = −1.3122, *p*-value = 0.09473).

For the test, on average, individuals performed better (Median = 4.0) using an LLM, compared to those who used Text (Median = 3.0). A Wilcoxon signed-rank test showed that this improvement is statistically significant (W = 30.0, Z = −2.04289, *p*-value = 0.02053).

Due to the exploratory nature of this study, this more lenient *p* threshold was adopted. Table 2 depicts how effect sizes indicated small associations for SCR (*r* = 0.25) and SCL (*r* = −0.25), and a moderate association for quiz performance (*r* = −0.49). Confidence intervals were wide, further reflecting limited power, and results should be interpreted as preliminary.

Table 3 depicts how for the SAM, on average, individuals have the same response.

Table 4 depicts running a Wilcoxon signed-rank test on the values.

As *p*-values from the Wilcoxon signed-rank test are all higher than the level of significance, there is insufficient evidence to conclude that there is any difference between the SAM results.

**Figure 8:**
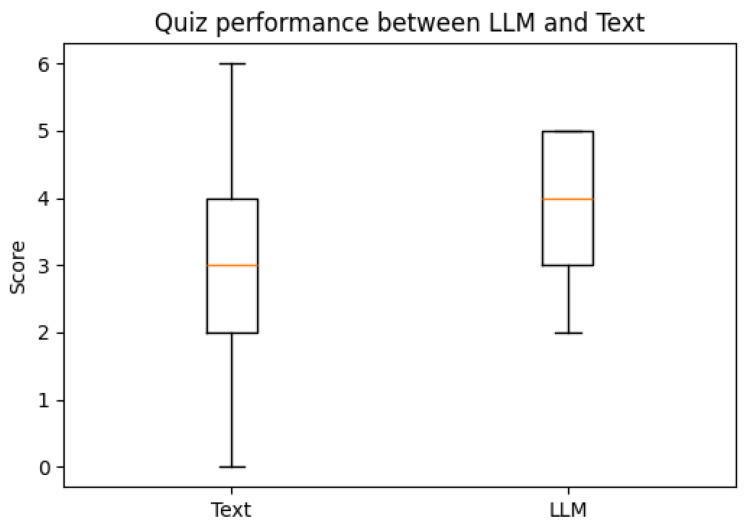
Quiz performance comparison between Text and LLM.

Interestingly, on average, it was found that quiz performance after learning via LLM was better than curated text, with LLM having a higher median and minimum score compared to curated text, as seen in Figure 8 below.

## 5. Discussion

Through this study, we have analyzed the differences in EDA between LLM and Text in learning, aiming to find differences in learners’ cognitive and emotional stresses.

Given that NSSCR increases with emotional arousal, we can infer that learning from the LLM results in a higher emotional arousal than learning from Text. However, compared to the self-reported emotional arousal from the SAM, there seems to be an inconsistency for any concrete conclusion. This can be due to the discrete values of the SAM scale, externalities during the experiments or variations in preconception, learning styles and disciplines (e.g., how students felt towards the topic of sustainability). Nonetheless, the inference of SCR and SCL (more positive emotional valence and lower cognitive load) follows the results of the study by Sun and Zhou (2024), which suggested that Gen-AI could be extremely beneficial for students’ learning [34]. Our results also provided empirical evidence, which is backed by another research by Wu and Yu (2024), which suggested that AI could enhance learning outcomes in the short term, for it can boost students’ confidence and motivation [35].

In contrast, there are studies that state otherwise. According to a study in 2025 by Gerlich on AI reliance and critical thinking, it was proposed that as people rely more on AI technology, they are less likely to engage in critical thinking, as they could resort to AI for cognitively straining tasks [5]. This behavior—referred to as cognitive offloading—is detrimental and ineffective in boosting learners’ efficacy. As for our results from the SAM, there was virtually no difference between any dimension of the sentiment scale, which indicates that there are no significant improvements that Generative AI brings in terms of learning new knowledge.

Furthermore, our results suggest that while there are differences in the students’ engagement and stress with educational content delivered between LLMs and curated text, the only statistically significant difference was emotional arousal (*p* = 0.094 via analysis of SCR) and cognitive load (*p* = 0.095 via analysis of SCL). Hence, one significant finding is that LLMs elicited more positive emotions while Text needed more memory from learners. Therefore, to further strengthen this effect, learners should aim for a higher degree of agency and active involvement with AI by using prompts that elicit active engagement (and thus, emotional response). For example, rather than asking for direct answers, learners can ask gen AI for more thought-provoking ideas that challenge their understanding of the topic. With a lower cognitive load, learners are less stressed and can therefore absorb information better. This corroborated the importance of utilizing AI correctly—careful scaffolding to provide learner agency and participation. While these are revealing insights with respect to observable differences in the effectiveness of Generative AI and curated text, with the overall landscape of our results, it is inconclusive whether Generative AI would be a superior alternative to curated text in terms of learning. Thus, we recommend that Generative AI should still be used in moderation and should not be the sole method that learners rely on.

To elaborate on this, our research supports the suggestions laid out by the UNESCO AI Competency Framework for Teachers. As Generative AI in our research has been shown to be only marginally better and have little to no additional perceived effectiveness in comparison to traditional means of learning, teachers’ role in education cannot be replaced, as they represent an accurate source of information, and Generative AI remains only a tool to complement existing methods of studying. There are also several other implications of the use of AI in teaching and learning. Firstly, the use of AI must be sustainable, and stakeholders must think critically before incorporating AI, for the over-reliance on AI can bear detrimental consequences on learners’ attributes. Furthermore, AI should be approached as a means to empower teachers and students’ roles in education. Teachers are still positioned as the main facilitators who guide students in discerning the knowledge given by AI outputs versus traditional texts or sources of information. This aligns with ensuring human agency and critical thinking skills remain central in learning. Finally, a balance should be struck between the roles of humans and AI in cultivating and teaching knowledge. This is because while AI can enhance accessibility and explain content in a personally tailored manner, the initial source of information should be curated carefully and accurately by a human. Overall, moving forward, the use of AI in education must ensure greater safety, accountability, and inclusivity. Additionally, teachers must also constantly keep up with the emerging capabilities of AI to effectively harness its power for education.

We readily acknowledge the following limitations to our approach. Firstly, the arbitrarily chosen level of significance of 0.1 for clustered and normal Wilcoxon signed-rank tests might still be too high to prove the statistical difference within the data of each data feature. Furthermore, one of the inherent weaknesses of EDA data is its low resolution, despite the ease of collecting data. Furthermore, while features extracted from EDA data can be reliable indicators of emotional arousal, it is less dependable as a means to analyze emotional valence. To mitigate this, the sensors were placed on the fingers of the non-dominant hand of each participant, in accordance with the protocols from previous studies [25,36]. We also assumed participants learned at a uniform pace, and the fixed time-windows within the procedure were a consequence of this assumption. We acknowledge this may have masked individual differences in engagement, effort, and emotional responses. Hence, in the future, alternative sources of data can be explored to complement existing ones, such as employing Electroencephalography (EEG) data or Facial Action Units (FAU). By using various sources of data and cross-checking the results of affective states and mental load, a more conclusive result can be obtained regarding the effectiveness of Generative AI compared to traditional learning means, such as curated text. We also acknowledge the shortcomings of our samples in terms of quantity, age, gender and nationality. Due to resource constraints, participants during the text-learning condition were not afforded access to a human learning partner; we acknowledge the asymmetry in conditions, but also draw parallels between self-study learning when no human pedagogues are present. Given the limited number of samples, age range (13–16) and the significant differences of cognitive, emotional and behavioral patterns across various age groups, the results can only be exemplary of this group of adolescent learners. In the future, these external factors will be explored for greater scope and robustness of information.

In future, as more advanced LLM models are trained and compositional learning is better demonstrated, we can expect to see more drastic changes in the effectiveness of Generative AI in facilitating students’ learning new knowledge. This is because future Generative AI models will be able to comprehend human language and images better, which further refines their response to the users’ prompt [37].

## 6. Conclusions

By way of tentative conclusion, with the rise in popularity of Generative AI as a tool to assist teaching and learning, our study contributes valuable empirical evidence to elucidate the degree of effectiveness of Generative AI compared to traditional methods of learning, such as curated text. This is especially crucial because ample, data-driven evidence is needed for teachers, policymakers, and other relevant stakeholders to make informed and grounded decisions regarding the planning, adoption, and implementation of Generative AI in education. Our findings are particularly significant in the current context, where there is growing interest in integrating AI tools into educational practices, often without sufficient critical evaluation of their actual benefits and limitations. As such, we aim to complement existing studies on Generative AI and education that employ observational and qualitative methods.

In our study, we employed EDA features and self-reported data as measures of learning effectiveness and conducted statistical analyses to draw meaningful observations. To summarize, we found that learning with a large language model (LLM) results in greater Skin Conductance Response (*p* = 0.09404), indicative of more positive emotional arousal, and lower Skin Conductance Level (*p* = 0.09473), suggesting reduced cognitive load, compared to curated texts. Moreover, our data revealed that learning with an LLM correlates with higher quiz performance (*p* = 0.02053), highlighting a potential benefit of Generative AI in enhancing test-based outcomes. However, as the rest of the features (NSSCR, TVSymp, and EDASymp) showed no statistically significant differences between learning via LLM and curated texts, our initially stated hypothesis was rejected.

Furthermore, concerning the results yielded by the SAM (self-reported data), participants generally perceived that LLMs did not significantly aid their learning compared to curated texts when acquiring new knowledge. Taken together with the rejected hypothesis, we conclude that while Generative AI may show slight advantages in specific areas, its overall benefit in learning new knowledge is marginal compared to traditional study methods like curated texts. Likewise, while the findings suggest that AI usage improves well-being, the methodology’s limitations—such as the non-representative sample and the specific context of the study—mean that caution should be exercised when applying these conclusions to broader populations or different settings. This suggests that Generative AI is not a definitive substitute for curated educational materials but rather a complementary tool that needs careful contextualization in learning environments.

Based on these caveats and results, we emphasize the importance of continuing research in this area to build a more comprehensive understanding of the effectiveness (or otherwise) of Generative AI in teaching and learning. Given that our work is an exploratory and preliminary study, future studies should aim to refine methodologies, incorporate diverse measures of learning outcomes, and explore how specific contexts, subjects, or demographics may influence the utility of Generative AI. With a strong foundation of empirical evidence, education stakeholders will be better equipped to integrate AI tools responsibly and effectively into teaching and learning practices.

## Figures and Tables

**Table 1:** Results of the clustered Wilcoxon signed-rank test and traditional Wilcoxon test for NSSCR, SCR, SCL, TVSymp and EDASymp.

Feature	Clustered Wilcoxon	Wilcoxon
NSSCR	Z = 0.2478, *p*-value = 0.4021	W = 3178.0, *p*-value = 0.5536
SCR	Z = 1.3163, *p*-value = 0.09404	W = 2497.0, *p*-value = 0.006494
SCL	Z = −1.3122, *p*-value = 0.09473	W = 2495.0, *p*-value = 0.006390
TVSymp	Z = 0.50823, *p*-value = 0.3056	W = 3017.0, *p*-value = 0.18508
EDASymp	Z = 0.29307, *p*-value = 0.3847	W = 3283.0, *p*-value = 0.54124

**Table 2:** Effect sizes and 95% Confidence interval for SCR, SCL and quiz results.

	Effect Size (r)	95% Confidence Interval
SCR	*r* = 0.25053	(−0.00784, 0.519)
SCL	*r* = −0.25104	(−0.615, −0.0338)
Quiz	*r* = −0.49123	(0.0, 2.0)

**Table 3:** Median score for self-reported data of the SAM.

Rows:	Median for Text:	Median for LLM:
Row 1 (valence)	7.0	7.0
Row 2 (arousal)	6.0	6.0
Row 3 (dominance)	6.0	6.0

**Table 4:** Results of the Wilcoxon signed-rank test for emotional valence, arousal and dominance.

Rows:	Results:
Row 1 (valence)	W = 156.0, z = −0.79943, *p*-value = 0.42404
Row 2 (arousal)	W = 182.5, z = −0.47158, *p*-value = 0.63723
Row 3 (dominance)	W = 174.0, z = −0.94686, *p*-value = 0.34371

**Figure A1:**
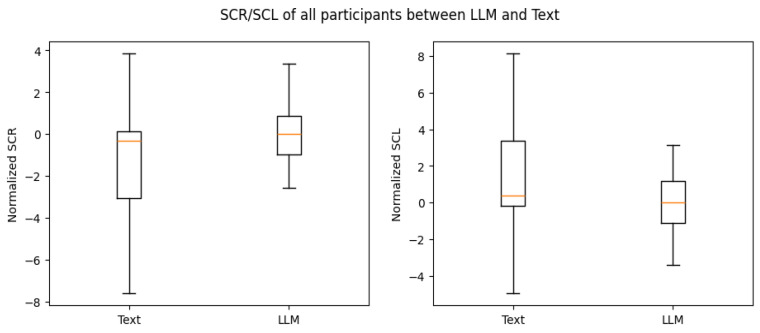
Box and whisker plot of SCR/SCL of participants between LLM and text.

## Data Availability

Data associated with this study are available upon reasonable request, due to privacy/ethical restrictions.

## Appendix A

Sustainability issues affect all of humanity, though the exact nature of the issues differs due to the context of each place. An example is climate change; a small tropical island state like Singapore is affected directly and indirectly by climate-related risks. Warming global temperatures see Singapore experiencing changes in weather patterns with more intense rainfall. Rising sea levels due to melting ice caps and the thermal expansion of seawater mean potential loss of low-lying land as well as floods. Events that occur in one part of the world have a ripple effect felt and seen by other parts of the world too, including Singapore. Singapore’s reliance on food imports, for instance, means that the resilience of farming in places like the Mekong Delta is of importance. Threats to agricultural areas such as droughts and saltwater incursion caused by climate change and changes to river systems due to dams and riverbed mining impact farmers who might decide to stop farming and move to cities in search of alternative livelihoods, an act that affects global and regional food supplies. There is an urgency to understand what is happening to other people in countries across the globe, as well as the need to search for solutions to the problems they face. These solutions may mean the need to help others mitigate and adapt to climate change even though they are not in Singapore, as all live in a hyper-connected world.

Concern for preserving the physical environment (e.g., protecting forests and nature spaces, as well as preventing pollution of the natural environment) begins as early as the late 1800s. However, in the 1970s and 1980s, environmental education gains traction, with the Belgrade Charter (UNESCO, 1975) recognizing that to conserve the physical environment, the social, cultural, and political dimensions must be addressed as well. Over time, “environmental education” changes to “sustainability education” as there is more recognition of the importance of integrating the complex inter-relationships among the physical environment and social, cultural, and political aspects of societies into environmental education. An international resolution adopted by the United Nations (UN), the “Decade of Education for Sustainable Development (2005–2014),” emphasizes the need to integrate sustainable development issues like climate change, biodiversity, and disaster risk reduction into all aspects of education and learning (UNESCO, 2005). In 2015, the UN adopts the Sustainable Development Goals (SDGs), which not only broaden the scope of sustainable development issues but also continue the emphasis on sustainable development through education (UN, 2015).

Schools play an important role in developing the knowledge and skills that young people need to participate in sustainability issues and work towards those UN sustainable development goals. Geography is a natural fit during UNESCO’s Decade of Education for Sustainable Development as it is a discipline that addresses issues like climate change, biodiversity, and disaster risk reduction. However, with the breadth of sustainable development goals today, all subjects have the capacity to tackle and engage students on sustainability issues. Science subjects engage students around the science of climate change, impacts of development, and climate change on ecologies. Social Studies is a key subject that educates students about governance. Sustainable development and climate issues involve governance and how individuals can engage with the state on these matters. Languages and Art subjects focus on how to communicate about sustainability issues too. A holistic and interdisciplinary approach to sustainability education is important, and the Ministry of Education’s Eco Stewardship Programme (ESP) serves as one of the important building blocks in this endeavour. The implementation of ESP in local educational institutes sees schools and institutes of higher learning integrating sustainable development into their curriculum, campus infrastructure, institutional culture and practices, as well as partnerships with the community.

Reflecting further on the future direction of sustainability education, the good work done in schools in this area is acknowledged. However, it is also important for school leaders and teachers to think ahead and develop more innovative pedagogies in their approach to sustainability education. If these issues are taught in a factual way, then students will treat them just like any other topic they are required to learn for assessment. Students should be encouraged to understand how people in other parts of the world are already impacted by issues like climate change. For example, how do people without air-conditioning or stable water supplies cope with heatwaves and droughts, and who are the people who are losing their homes to rising sea levels? It is also important that students are provided with positive examples of what people are doing to overcome these problems. These could be in the form of innovations, community initiatives, and partnerships among individuals, businesses, and governments. Students should realize that the problems the world faces are not necessarily insurmountable, and that they have the power to make informed decisions and take individual and collective action. It would be best if students are empowered to apply these insights to issues they themselves have witnessed and want to address.

Hint: Below are some topics that will be tested

- Impacts of climate change
- Effects of the impacts of climate change, e.g., supply chain disruptions
- How to achieve sustainability
- Details about specific initiatives mentioned
  ○ UN Initiatives
  ○ MOE Initiatives

## Appendix B

You are now a teaching assistant, preparing the student for a test. Explain the concept that will be given to you.

----------------------

# Objective

You will be given information on the topic below, as well as the scope of the test. Start a chat with the student. You should start with a brief overview on the topic, as well as what is tested. Cover all aspects of the topic that may be tested. You may let the students know what is tested, but do not reveal the information given verbatim.

----------------------

# Style

Friendly, elaborative, example-driven

----------------------

# Tone

Warm and inviting

----------------------

# Audience

10 to 12 year old Filipino students, in Singapore for a learning activity.

=====================

# Background information

Sustainability issues affect all of humanity, though the exact nature of the issues differs due to the context of each place. An example is climate change; a small tropical island state like Singapore is affected directly and indirectly by climate-related risks. Warming global temperatures see Singapore experiencing changes in weather patterns with more intense rainfall. Rising sea levels due to melting ice caps and the thermal expansion of seawater mean potential loss of low-lying land as well as floods. Events that occur in one part of the world have a ripple effect felt and seen by other parts of the world too, including Singapore. Singapore’s reliance on food imports, for instance, means that the resilience of farming in places like the Mekong Delta is of importance. Threats to agricultural areas such as droughts and saltwater incursion caused by climate change and changes to river systems due to dams and riverbed mining impact farmers who might decide to stop farming and move to cities in search of alternative livelihoods, an act that affects global and regional food supplies. There is an urgency to understand what is happening to other people in countries across the globe, as well as the need to search for solutions to the problems they face. These solutions may mean the need to help others mitigate and adapt to climate change even though they are not in Singapore, as all live in a hyper-connected world.

Concern for preserving the physical environment (e.g., protecting forests and nature spaces, as well as preventing pollution of the natural environment) begins as early as the late 1800s. However, in the 1970s and 1980s, environmental education gains traction, with the Belgrade Charter (UNESCO, 1975) recognizing that to conserve the physical environment, the social, cultural, and political dimensions must be addressed as well. Over time, “environmental education” changes to “sustainability education” as there is more recognition of the importance of integrating the complex inter-relationships among the physical environment and social, cultural, and political aspects of societies into environmental education. An international resolution adopted by the United Nations (UN), the “Decade of Education for Sustainable Development (2005–2014),” emphasizes the need to integrate sustainable development issues like climate change, biodiversity, and disaster risk reduction into all aspects of education and learning (UNESCO, 2005). In 2015, the UN adopts the Sustainable Development Goals (SDGs), which not only broaden the scope of sustainable development issues but also continue the emphasis on sustainable development through education (UN, 2015).

Schools play an important role in developing the knowledge and skills that young people need to participate in sustainability issues and work towards those UN sustainable development goals. Geography is a natural fit during UNESCO’s Decade of Education for Sustainable Development as it is a discipline that addresses issues like climate change, biodiversity, and disaster risk reduction. However, with the breadth of sustainable development goals today, all subjects have the capacity to tackle and engage students on sustainability issues. Science subjects engage students around the science of climate change, impacts of development, and climate change on ecologies. Social Studies is a key subject that educates students about governance. Sustainable development and climate issues involve governance and how individuals can engage with the state on these matters. Languages and Art subjects focus on how to communicate about sustainability issues too. A holistic and interdisciplinary approach to sustainability education is important, and the Ministry of Education’s Eco Stewardship Programme (ESP) serves as one of the important building blocks in this endeavour. The implementation of ESP in local educational institutes sees schools and institutes of higher learning integrating sustainable development into their curriculum, campus infrastructure, institutional culture and practices, as well as partnerships with the community.

Reflecting further on the future direction of sustainability education, the good work done in schools in this area is acknowledged. However, it is also important for school leaders and teachers to think ahead and develop more innovative pedagogies in their approach to sustainability education. If these issues are taught in a factual way, then students will treat them just like any other topic they are required to learn for assessment. Students should be encouraged to understand how people in other parts of the world are already impacted by issues like climate change. For example, how do people without air-conditioning or stable water supplies cope with heatwaves and droughts, and who are the people who are losing their homes to rising sea levels? It is also important that students are provided with positive examples of what people are doing to overcome these problems. These could be in the form of innovations, community initiatives, and partnerships among individuals, businesses, and governments. Students should realize that the problems the world faces are not necessarily insurmountable, and that they have the power to make informed decisions and take individual and collective action. It would be best if students are empowered to apply these insights to issues they themselves have witnessed and want to address.

# Test information

The test will have less than 10 MCQ questions. The topics tested include:

- Impacts of climate change
- Effects of the impacts of climate change, e.g., supply chain disruptions
- How to achieve sustainability
- Details about specific initiatives mentioned (UN Initiatives, MOE Initiatives)

IMPT: Don’t do markdown formatting in your responses
